# Biallelic disruption of DDX41 activity is associated with distinct genomic and immunophenotypic hallmarks in acute leukemia

**DOI:** 10.3389/fonc.2023.1153082

**Published:** 2023-06-26

**Authors:** Anne Tierens, Elizabeth Kagotho, Satoru Shinriki, Andrew Seto, Adam C. Smith, Melanie Care, Dawn Maze, Hassan Sibai, Karen W. Yee, Andre C. Schuh, Dennis Dong Hwan Kim, Vikas Gupta, Mark D. Minden, Hirotaka Matsui, José-Mario Capo-Chichi

**Affiliations:** ^1^ Department of Laboratory Medicine and Pathobiology, University of Toronto, Toronto, ON, Canada; ^2^ Department of Pathology and Laboratory Medicine, Aga Khan University Hospital, Nairobi, Kenya; ^3^ Department of Molecular Laboratory Medicine, Faculty of Life Sciences, Kumamoto University, Kumamoto, Japan; ^4^ Division of Clinical Laboratory Genetics, Laboratory Medicine Program, University Health Network, Toronto, ON, Canada; ^5^ Department of Medicine Medical Oncology and Hematology, University of Toronto, Princess Margaret Cancer Centre, Toronto, ON, Canada

**Keywords:** acute leukemia, myelodysplastic syndrome, DDX41 mutations, biallelic, clinical pathology

## Abstract

**Introduction:**

Inherited *DDX41* mutations cause familial predisposition to hematologic malignancies including acute myeloid leukemia (AML) and myelodysplastic syndromes (MDS), with the majority of DDX41 mutated MDS/AMLs described to date harboring germline *DDX41* and co-occurring somatic *DDX41* variants. DDX41-AMLs were shown to share distinguishing clinical features such as a late AML onset and an indolent disease associated with a favorable outcome. However, genotype-phenotype correlation in DDX41-MDS/AMLs remain poorly understood.

**Methods:**

Here, we studied the genetic profile, bone marrow morphology and immunophenotype of 51 patients with DDX41 mutations. We further assessed the functional impact of ten previously uncharacterized *DDX41* variants of uncertain significance.

**Results:**

Our results demonstrate that MDS/AML cases harboring two *DDX41* variants share specific clinicopathologic hallmarks that are not seen in other patients with monoallelic *DDX41* related hematologic malignancies. We further showed that the features seen in these individuals with two *DDX41* variants were concordant with biallelic *DDX41* disruption.

**Discussion:**

Here, we expand on previous clinicopathologic findings on *DDX41* mutated hematologic malignancies. Functional analyses conducted in this study unraveled previously uncharacterized *DDX41* alleles and further illustrate the implication of biallelic disruption in the pathophysiology of this distinct AML entity.

## Introduction

Germline mutations in *DDX41* cause familial predisposition to myeloid malignancies including acute myeloid leukemia (AML) and myelodysplastic syndromes (MDS) (1–9). Single acquired *DDX41* variants on their own were not identified as major contributors to the pathophysiology of AML, MDS and myeloproliferative neoplasms (MPN). However, not uncommon is the co-occurrence of additional somatic *DDX41* variants in individuals with a germline *DDX41* mutation (1–9). Such AML presenting with multiple *DDX41* variants share distinguishing features, suggesting that the presence of germline *DDX41* and acquired *DDX41* mutation(s) defines a specific AML subgroup. For example, DDX41-AML is a late onset disease with an indolent presentation and a favorable outcome; features that contrast with other types of inherited or acquired AMLs (1–9). Several somatic and germline *DDX41* mutations have been reported to date. The deleterious potential of loss of function variants (i.e frameshift, nonsense and splicing) can be easily inferred. However, the functional impact of numerous missense, in-frame and start loss variants identified in *DDX41* has not been assayed. Functional characterization of such *DDX41* variants of uncertain significance (VUS) is of great clinical value. For instance, elucidating the role of a previously uncharacterized germline *DDX41* variant is of crucial importance to genetic counselling of an affected carrier as well as family risk relatives, given considerations of familial predisposition to myeloid malignancies.

Here, we sought to analyze the genomic architecture, bone marrow morphology as well as immunophenotypes of patients with *DDX41* mutated AML, MDS and MPN to explore possible genotype–phenotype correlations between individuals with these *DDX41* related hematologic malignancies. To this effect, we further investigated the impact of ten previously reported, yet uncharacterized *DDX41* VUS. Our results expand on previous reports on *DDX41* related malignancies. Unique to this study, we provide supportive *in-vivo* and *in vitro* evidence that biallelic disruption of *DDX41* is associated with unique clinicopathologic features observed in this distinct AML entity.

## Material and methods

### Study cohort

The study cohort constituted of 1930 patients with myeloid malignancies diagnosed at the Princess Margaret Centre. DNA was extracted from peripheral blood (PB) or bone marrow (BM) samples of myeloid malignancies cases studied herein. The study was approved by the University Health Network Research Ethics Board.

### Cytogenetics

G-banding analyses were conducted on all cases with analyzable metaphases. Where G-banding analyses were unsuccessful, reflex FISH testing was performed to rule out possible deletions on chromosome 5 where *DDX41* is located.

### Next-generation sequencing and variant analysis

Genomic DNA was sheared and library capture by hybridization was performed by using a pool of custom probes targeting hotspot sequences or exonic coding regions and flanking intronic sequences of 49 genes considered clinically relevant in hematologic malignancies (**Table S1**). The gene targets include the entire consensus coding DNA regions of *DDX41*. Libraries were visualized (TapeStation, Agilent Technologies), and sequencing was performed by using the Illumina Next-Generation Sequencing platform (paired-end sequencing, MiSeq version 2). Bioinformatics analyses included alignment to the human genome reference build GRCh37/hg19 using the Burrows-Wheeler Alignment, processing and quality metrics using the Genome Analysis Toolkit and Picard, and variant calling using VarScan2. Variants not meeting laboratory-defined quality metrics (read depth <100, population frequency >1% in the gnomAD database, or VAF <2%) were removed from further analysis.

Variant filtering was performed by using the Alissa Clinical Informatics Platform (Agilent Technologies). Variants lying outside of coding sequences or canonical splice sites were filtered out, along with synonymous alterations. The remaining variants were classified as pathogenic/likely pathogenic or variants of uncertain significance following the CAP/AMP/ASCO somatic variants classification guidelines (10). Variant annotation was conducted as previously described (11–13). Briefly, evidence were gathered from published literature (eg, PubMed: ncbi.nlm.nih.gov/pubmed/), as well as cancer-wide (eg, COSMIC: cancer.sanger.ac.uk/cosmic) and gene-specific (eg. TP53 IARC: http://p53.iarc.fr/) repositories. Pathogenic (Tier-1) or likely pathogenic (Tier-2) variants included the following: (1) variants affecting hotspot codons (eg. p.R525H in *DDX41*); (2) variants occurring at critical gene regions or protein functional domains in which other deleterious variants have been reported in myeloid neoplasms (eg. truncating variants in the DEAD box of DDX41); and (3) variants predicted to disrupt the normal activity of the encoded protein (eg. start loss variant in *DDX41*). Variants not meeting any of the aforementioned criteria were classified as variants of uncertain significance (Tier-3).

### Morphologic and immunophenotypic analyses

For immunphenotyping, heparin anticoagulated bone marrow aspirates were processed according to the Euroflow standard procedures for sample preparation, staining and acquisition as previously described (14). Bone marrow smears were stained with Wright Giemsa. Bone marrow trephine were fixed in B+ decalcified and stained with Hematoxylin and Eosin. Acquisition was performed on Lyric Instruments (Becton Dickenson (BD) Biosciences, San Jose, CA, USA). The files were analyzed using Infinicyt v.2.1 (Cytognos TM, Salamanca, Spain).

### Construction of plasmids

DDX41 variant expression vectors (p.Met1?, p.Ala11Thr, p.Met155Ile p.Pro213Arg, p.Gly218Asp, p.Thr320Ile, p.Glu345Lys, p.Ala346Pro, p.Ser363del, p.Arg479Trp, p.Arg525His and p.Asp570Gly) were constructed using the KOD-Plus-Mutagenesis Kit (Toyobo Co., Ltd., Tokyo, Japan) based on the pcDNA4 myc-His plasmid inserted with wild-type (WT) DDX41 (15). The primers used for the construction are shown in **Table S2**.

### RNA interference *via* shRNA

The pLKO.1 puro vector inserted with an shRNA targeting the 3’UTR region of the DDX41 gene (shDDX41-UTR) was transfected into HEK293FT cells together with the psPAX2/pMD2G packaging plasmids to produce lentivirus for DDX41 knockdown. The target sequence for DDX41 knockdown is CCTGTGCTCTTCAGAATTA. pLKO.1-scramble shRNA (shScr), a gift of David Sabatini (Addgene plasmid #1864), was used for control (16). The virus in the culture supernatant was infected to HEK293T cells, and the knockdown was confirmed by western blot analysis.

### Cell viability assay

DDX41-expressing plasmid (WT, p.R525H and other 11 variants) or an empty plasmid for a negative control were transfected into HEK293T cells using Polyethylenimine MAX (Polysciences, Warrington, PA, USA) according to the manufacturer’s instructions. The culture medium was replaced with fresh medium 8 hours after the transfection, after which the cells were infected with the lentivirus for DDX41 knockdown as described above. The number of viable cells was counted by trypan blue staining on day 6 after the infection. A schematic illustration of the experimental procedure was shown in **Figure S1**.

### Cell cycle analysis

The cells were washed with PBS and fixed overnight in 70% ethanol chilled at -20°C. Fixed cells were washed with PBS and incubated with propidium iodide (50 μg/mL) and RNase A (10 μg/mL) for 20 min at room temperature. After filtration, the cell suspensions were analyzed using FACSVerse (BD Biosciences, San Jose, CA, USA).

### Data analysis and display of experimental results

For cell viability assay and cell cycle analysis, the experiments were performed in triplicate. Due to the limited sample numbers in each assay, all individual data points for these experiments were plotted alongside error bars.

## Results

### Study cohort

We have sequenced the tumor of 1930 patients with myeloid malignancies including AML, MDS and MPN using a targeted myeloid next-generation sequencing (NGS) panel (**Table S1**). In total, we have identified 51 cases (2.6%) with at least one mutation in *DDX41* (**Table 1**). Our study cohort consist of 13 DDX41-MPNs and 38 DDX41-AMLs. Cytogenetic analyses conducted in parallel to NGS revealed a deletion chromosome 5q35 encompassing *DDX41* in one patient (case M22) that also harbored a *DDX41* point mutation (**Table 1**).

**Table 1:** Genomics findings in myeloid cases with mutations in DDX41.

Case	Age	Sex	Blast %	Diagnosis	Cytogenetic analyses	DDX41 Variants		
						VAF>45%	VAF<33%	VAF<33%
S1	68	F	N/A	MPN	N/A	p.V303M: 50%		
S2	49	M	N/A	MPN	N/A	p.M155I: 48.6%		
S3	21	M	N/A	MPN	N/A	p.I176T: 50.2%		
S4	75	M	N/A	MPN	N/A	p.R479W: 46.6%		
S5	87	F	N/A	MPN	46,XX[20]	c.936C>T, p.?: 52%		
S6	77	M	N/A	MPN	46,XY[24]	p.G610S: 49.3%		
S7	54	F	N/A	MPN	N/A	p.D73E: 49.7%		
S8	61	M	N/A	MPN	N/A		p.E15K: 5.5%	
S9	56	F	N/A	MPN	46,XX,del(13)(q12q14)[20]	p.K331M: 48.7%		
S10	65	M	N/A	MPN	46,XY[20]	p.V303M: 49.9%		
S11	75	F	N/A	MPN	N/A	p.Y33H: 50.7%		
S12	79	M	N/A	MPN	N/A	p.R339L: 49.3%		
S13	30	F	N/A	MPN	N/A	p.Q48*: 51.4%		
S14	39	M	79	APL	N/A	p.R293C: 49.5%		
S15	61	M	24	AML	46,XY,2~16dmin[11]/46,XY[8], nuc ish(MYCx6~amp)(5’MYC con 3’MYCx6~amp)[135/200]	p.A11T: 51.9%		
S16	77	F	26	AML-MRC	46,XY[20]		p.L425P: 10.8%	
S17	35	M	28	AML	45,XY,der(13;22)(q10;q10)[11]/ 46,XY,t(10;11)(q22;q23), der(13;22)(q10;q10),+22[9]		p.I142Sfs*12: 6.3%	
S18	54	M	63	AML	46,XY,t(4;12)(q12;p13)[22]	p.E476*: 51%		
S19	73	F	85	AML	46,XX[22]	p.M155I: 49.6%		
S20	38	M	78	AML	46,XY[20]	p.R164W: 52.2%		
S21	42	M	93	AML	46,XY[20]	p.R164W: 50.2%		
S22	83	M	16	AML	46,XY[20]	p.V412I: 48.7%		
S23	79	M	16	AML	FISH Del(5q)/-5 Negative	p.G218D: 51.6%		
S24	66	M	>30	AML-NOS	46,XY[20]	p.R159*: 50.2%		
S25	79	M	22	AML-MRC	FISH Del(5q)/-5 Negative	p.R159*: 50.4%		
S26	21	M	5	MDS-U	46,XY[20]	p.E2A: 45.9%		
S27	74	M	24	AML-MRC	45,XY,-7[20]	p.K187R: 49.0%		
S28	70	F	21	AML	FISH Del(5q)/-5 Negative	p.Lys381*: 51.5%		
S29	78	F	15	t-AML, PCN	47,XX,+8[6]/47,idem,del(6) (q13q23)[2]/46,XX[17]	p.R525H: 78.0%		
M1	64	M	22	AML	FISH Del(5q)/-5 Negative	p.D140Gfs*2: 63%	p.R525H: 6.3%	
M2	60	M	35-40	AML	FISH Del(5q)/-5 Negative	p.I142Sfs*12: 47.2%	p.R525H: 6.1%	
M3	67	M	32	AML	45,X,-Y[6]/46,XY[16]	p.Lys381*: 53.1%	p.R525H: 11.2%	
M4	76	M	29	AML	FISH Del(5q)/-5 Negative	p.M316Dfs*31: 51.6%	p.R525H: 5.6%	
M5	69	M	23	AML, PCN	FISH Del(5q)/-5 Negative	p.T529Rfs*12: 45.4%	p.R525H: 10.0%	p.P321L: 4.6%
M6	75	M	39	AML-MRC	46,XY[22]	c.435-1G>T, p.?: 50.7%^Ψ^	p.D570G: 4.9%	p.P321L: 33.0%
M7	75	M	38	AML-MRC	44,XY,add(2)(p2?3),del(4)(q25),der(6)t(6;14)(p23;q11.2),del(7)(q22q22),add(10)(p13),del(12)(q14),14,22[3]/45,idem,+8[3]/46,XY[11]	c.935+4A>T, p.?: 48.2%	p.R525H: 16.2%	
M8	68	M	23	AML	46,XY[11]	c.434+1G>C, p.?: 49.1%^Ψ^	p.R525H: 4.0%	
M9	54	M	24	AML	46,XY[20]	p.Q329Rfs*7: 46.7%^Ψ^	p.R525H: 3.2%	
M10	91	M	30	AML-MRC	46,XY,del(4)(q12),add(7)(p22),del(13) (q32),-18,add(18)(q23), +mar[cp4]/46,XY[17]		p.R525H: 15.9%	p.F535Nfs*6: 12.4%
M11	73	M	10-15	MDS-EB2	46,XY[20]	c.1231-3C>G, p.?: 49.8%	p.R525H: 4.5%	
M12	70	M	70	AML-NOS	46,XY,del(20)(q11.2q13.3)[8]/46,XY[17]	p.L283Cfs*21: 54.6%	p.R525H: 31.8%	
M13	71	F	15-20	AML-NOS	46,XX[20]	p.Met1?: 47.9%	p.R525H: 6.5%	
M14	89	M	31	AML-NOS	46,XY[21]	p.S363del: 47.5%	p.R525H: 9.4%	
M15	74	M	34	AML	46,XY[13]	p.G218D: 50.7%	p.R525H: 7.0%	
M16	79	M	40	AML	46,XY[20]	p.P213R: 49%	p.E345D: 3.6%	
M17	61	M	27	AML-NOS	45,X,-Y[4]/46,XY[16]	p.E345K: 48.6%	p.A346P: 4.4%	
M18	71	M	11	MDS-EB2	46,XY[23]	p.T320I: 49.%	p.G530D: 4.8%	
M19	69	F	30-40	AML	FISH Del(5q)/-5 Negative	p.Met1?: 50%^Ψ^	p.G402W: 6.4%	
M20	82	F	18	AML	FISH Del(5q)/-5 Negative		p.R525H: 7%	p.G587C: 5.9%
M21	64	M	5-7	MDS	FISH Del(5q)/-5 Negative	p.Y516C: 49.8%	p.A376T: 4.2%	
M22	65	M	19	AML	44,XY,del(5)(q11.2),add(7)(q11.2), add(12)(p11.2),add(15)(q?21),-17, -19[cp13]/46,XY[7]		p.R369Q: 22.4%	

^Ψ^ Confirmed germline in skin fibroblasts. N/A, Not available.

All the DDX41-MPNs (cases S1-S13) harbored a single *DDX41* variant (DDX41-s). The DDX41-AMLs constituted of 16 DDX41-s (cases S14-S29), as well as 22 cases (cases M1-M22) with multiple alterations in *DDX41* (DDX41-m). The study cohort composed of 13 females and 38 males (**Table 1**). DDX41-AMLs were predominantly males (DDX41-s AMLs = 12 or 75%, DDX41-m AMLs = 18 or 82%) compared to DDX41-MPNs (7 or 54%). DDX41-m presented with AML at older age (average = 71, minimum = 54, maximum = 91) compared to the DDX41-s AMLs (average = 61, minimum = 21, maximum = 83) and the DDX41-s MPNs (average = 61, minimum = 21, maximum = 87).

### Genomic features of *DDX41* mutated patients

We identified patients with one (n=29), two (n=20) or three (n=2) genetic lesions (i.e DNA point mutations and chromosome deletion) in *DDX41*. Variants detected in *DDX41* were distributed in two distinct clusters of variant allele frequency (VAF) with germline-like variants having a VAF >45% (average: 50%, maximum: 63%) and somatic variants VAF <33% (average: 9.4%, minimum: 3.2%). Repartition of variants within these VAF clusters applied to all the *DDX41* cases analyzed herein, except for an outlier (case S29) harboring the somatic hotspot p.R252H variant at 78%. Germline variants were confirmed by sequencing skin fibroblasts, where possible (**Table 1**).

**Figure 1:**
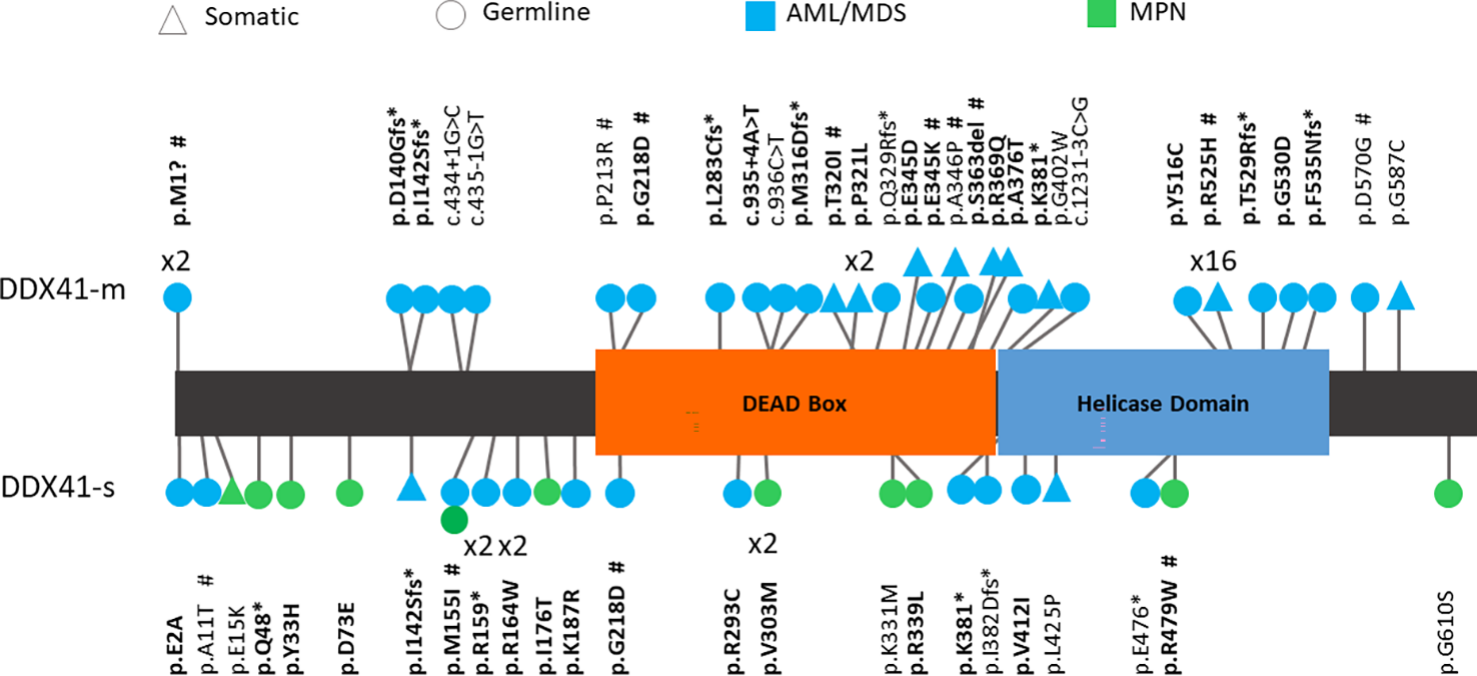
Schematic representation of variants detected in DDX41. Bold – Variants previously reported. # - Variant functionally assessed in this study.

In total we identified 74 *DDX41* variants including 44 germline-like and 30 somatic. More than half (74%) of these variants (germline = 33, somatic = 22) have been previously reported in a DDX41-mutated hematologic malignancy or were seen in more than one *DDX41* mutated patient from this study cohort (**Table 1**, **Figure 1**). The most common *DDX41* variants was the hotspot p.R525H (n=16).

Suspicious germline-like variants consisted of 24 missenses, 2 start-losses, 1 in-frame, 6 nonsenses, 6 frameshifts and 5 splicing variants (**Figure 1**). Somatic variants included 28 missenses and 2 frameshift variants. The 19 loss-of function (start loss, nonsense, frameshift and splicing) germline *DDX41* variants were classified as likely pathogenic variants using the ACMG germline variant classification criteria (17). However, the remaining 25 germline variants (missense and in-frame) were considered as variants of uncertain significance (VUS) due to the lack of evidence of their impact on the encoded protein. Previously, we showed that the somatic hotspot p.R525H variant cannot rescue the growth of hematopoietic cells in which endogenous *DDX41* had been eliminated (15). Based on this functional evidence, the p.R525H hotspot was determined to be a Tier-1 (pathogenic) variant using the AMP/CAP/ASCO somatic variant classification scheme (10). When applying the same scheme to other somatic *DDX41* variants, the 2 frameshifts were interpreted as Tier-2 (likely pathogenic) variants and 12 previously uncharacterized missense somatic variants were determined to be Tier-3 (VUS). In summary, of the 74 *DDX41* variants identified herein, half (germline and somatic) were classified as pathogenic or likely pathogenic variants and the other half including 25 germline and 12 somatic were VUS.

The majority of variants identified in the DDX41-s were of different nature and were located elsewhere in the protein than those seen in the DDX41-m. For instance, 12 out of 16 (75%) of the DDX41-s harbored a single missense VUS in non-catalytic regions (N- and C-terminal ends) of DDX41. In contrast, such missense VUS where only identified in 2 out of 8 (25%) of the DDX41-m. Instead 25 out of the 39 variants (64%) detected in the DDX41-m, were disruptive variants affecting the catalytic core DEAD box and (**Figure 1**). These deleterious DDX41-m variants include the hotspot p.R525H (n=16) and 9 additional loss-of-function (nonsense, frameshift, splicing variants).

### Co-occurrence of myeloid genes variants

**Figure 2:**
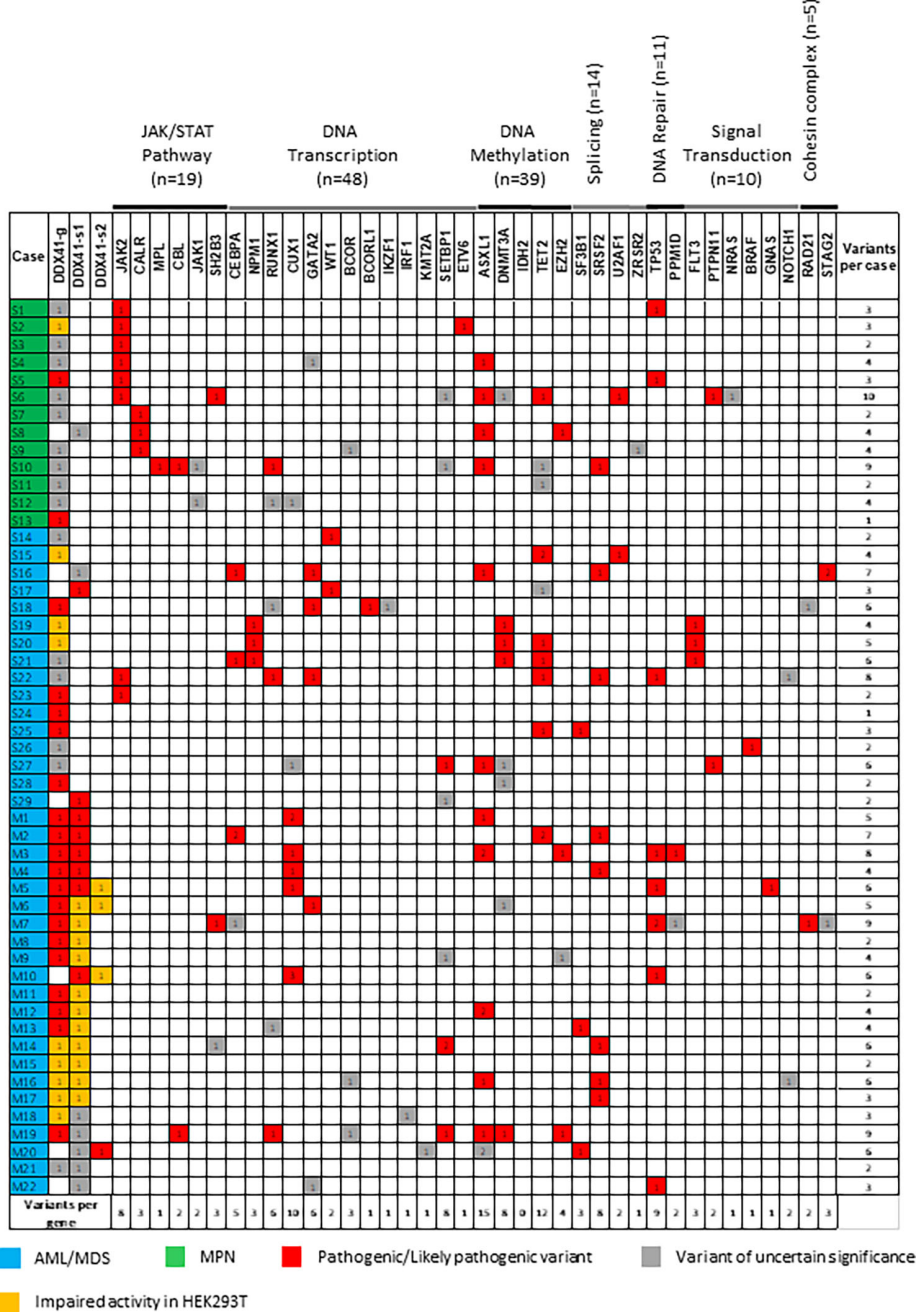
Co-occurring variants in DDX41 mutated patients. DDX41-g. DDX41 germline. DDX41-s. DDX41 somatic.

NGS detected additional variants in myeloid genes in the DDX41-MPNs and DDX41-AMLs (**Figure 2**). These co-occurring variants were predominantly seen in *ASXL1* (n=15), *TET2* (n=12), *CUX1* (n=10) and *TP53* (n=9). In aggregate, DNA transcription (n=54) and DNA methylation (n=43) genes accumulated the most variants. Overall, DDX41-s MPNs (minimum: 1, maximum: 10, average: 4), DDX41-s AMLs (minimum: 1, maximum: 8, average: 4) and DDX41-m AMLs (minimum: 2, maximum: 9, average: 5) shared similar numbers of co-occurring myeloid variants. However, variants affecting myeloid defining biomarkers were more frequently seen in the DDX41s-MPNs (*JAK2 =* 6, *CALR* = 3, *MPL* =1) and the DDX41-s AMLs (*CEBPA* biallelic =2, *NPM1* = 3, *RUNX1 =* 2) compared to the DDX41-m AMLs (*RUNX1 =* 1, **Figure 2**) (18). Furthermore, molecular and cytogenetics tests conducted in parallel to NGS identified other abnormalities (PML-RARA fusion =1, double minute chromosomes with *MYC* amplification = 1, *KMT2A* rearrangement =1, *ETV6* rearrangement =1) that could explain the origin of AML in 4 additional DDX41-s AMLs (19–21). In summary, the origin of MPN could be associated to the presence of a co-occurring variant and not a *DDX41* mutation, in 10 out of 13 (77%) of the DDX41-MPNs. Similarly, the etiology of AML could be linked to a genetic lesion not impacting *DDX41* in 11 out of 18 (61%) of the DDX41-s. However, such myeloid defining alterations were not unraveled in the 22 DDX41-m studied herein (**Figure 2**).

### Functional characterization of *DDX41* VUS

NGS identified 37 variants (25 germline and 12 somatic) variants that were determined to be VUS following ACMG, AMP, CAP, ASCO variants classifications schemes. More than half of these VUS (n =28) have been reported in association to *DDX41* mutated hematologic malignancies, but were never characterized. Here we sought to investigate the impact of 10 (8 germline and 2 somatic) of these VUS in HEK293T cells (**Table 1**, **Figure 1**). In selecting those 10 variants for functional assessment, priority was given to variants meeting the following criteria: (a) frequency of variant in *DDX41* mutated malignancies and (b) location of variant within the encoded DDX41 protein. As such, we retained 1 in-frame and 9 missenses variants for functional analyses conducted in HEK293T cells.

**Figure 3:**
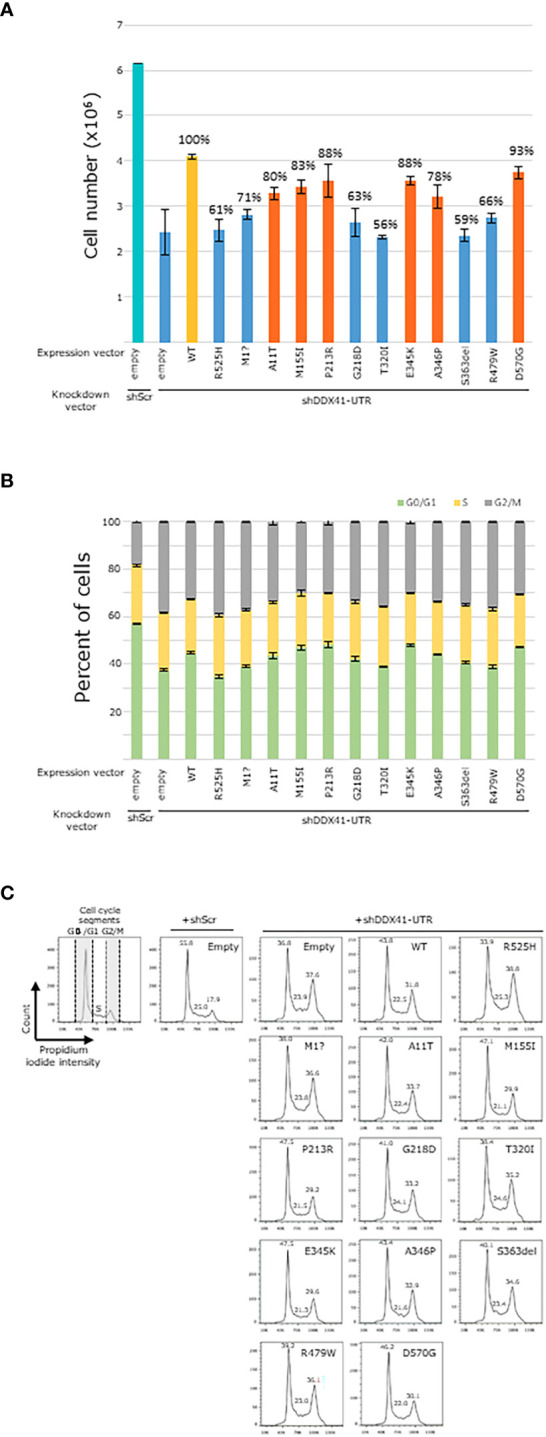
Functional characterization of DDX41 variants in HEK293T cells. **(A)**. Representative images of HEK293T cell count measurements performed in triplicate are shown. Percentage of cellular growth compared to HEK293T cells transduced with wild type *DDX41* is included on top of histograms. Blue. Variants showing 75% or fewer HEK293T cells when compared to HEK293T cells transduced with wild type *DDX41*. Orange. Variants showing greater than 75% of HEK293T cells when compared to HEK293T cells transduced with wild type *DDX41*. **(B)**. Plots indicate G0/G1 (green), S (yellow) and G2/M (gray) phases of all individual data points. **(C)**. Impact of selected DDX41 variants on cell cycle kinetics in HEK293T *DDX41* knockdown model. Values of each cell cycle phase are indicated in the histogram.

We have previously demonstrated that the hotspot p.R525H variant is a deleterious variant as this variant failed to rescue the cellular growth of hematopoietic cells in which endogenous *DDX41* had been eliminated (15). Here, we investigated the impact of *DDX41* mutants on the proliferation of HEK293T cells, within which the endogenous *DDX41* expression was repressed using a 3’UTR shRNA. First, we confirmed that consistent with previous results, p.R525H also failed to restore cellular growth in which endogenous *DDX41* was suppressed as elicited by 61% cells recovered compared to HEK293T cells transduced with wild-type *DDX41* (**Figure 3A**). We have previously showed that *DDX41* deficient hematopoietic cells can progress to the S phase of the cell cycle, albeit with mild DNA replication. However, these cells are stalled in the G2/M phase just before entering mitosis and arrest in the G2/M phase correlates with impaired cellular proliferation in these *DDX41* deficient cells (22). Here, we observed that the p.R525H mutant also distorted normal cell cycle of HEK293T cells in which endogenous *DDX41* was suppressed with fewer cells observed in G0/G1 (33.9 versus 43.8) and more cells detected in the S (25.3 versus 22.5) and G2/M (38.8 versus 31.8) phases compared to HEK293T cells transduced with wild-type *DDX41* (**Figures 3B, C**). Accumulation of cells in the G2/M phase is reminiscent of previous observations made in *DDX41* deficient hematopoietic cells (22).

To further validate the use of the HEK293T paradigm for our functional analyses, we also showed that another common germline *DDX41* allele, the start loss variant p.Met1?, elicited similar deficits on HEK293T proliferation (71% cells recovered compared to wild-type *DDX41*) and cell cycle (number of HEK293T cells in G0/G1: 38, S: 23.8 and G2/M: 36.6; **Figure 3**). Thus, the p.R525H and p.Met1? alleles show dysfunction in the HEK293T paradigm. Findings of disruption of *DDX41* by the p.Met1? allele is corroborated by previous studies showing that p.Met1? abrogates normal protein translation in HEK293T cells resulting in a shortened protein product with altered cellular localization (2).

Based on these observations, we assessed the ability of the ten *DDX41* VUS to impact the proliferation as well as cell cycle of HEK293T cells (**Figure 3**). Four variants (p.G218D, p.T320I, p.S363del and p.R479W) also disrupted cellular growth in HEK293T to comparable levels (between 56% and 66% of cells recovered compared to wild-type *DDX41*) to those observed with p.R525H and p.Met1? (blue bars, **Figure 3A**). Six other variants (p.A11T, p.M155I, p.P213R, p.E345K, p.A346P and p.D570G) also failed to restore cellular growth of HEK293T cells; although between 78% and 93% of cells were recovered compared to wild-type *DDX41* (orange bars, **Figure 3A**). Our results showed that the same *DDX41* mutants also impaired normal cell cycle in HEK293T cells. Although, cell cycle kinetics varied between the *DDX41* mutants investigated herein; overall, all the mutant alleles showed a distortion of the cell cycle with an accumulation of cells in G2/M which are reminiscent of previous observations made in hematopoietic cells (22). In summary, we showed that the ten *DDX41* variants analyzed herein impaired cellular proliferation and normal cell cycle in HEK293T cells suggesting that these are deleterious *DDX41* alleles.

### Bone marrow features of DDX41-m AMLs

**Figure 4:**
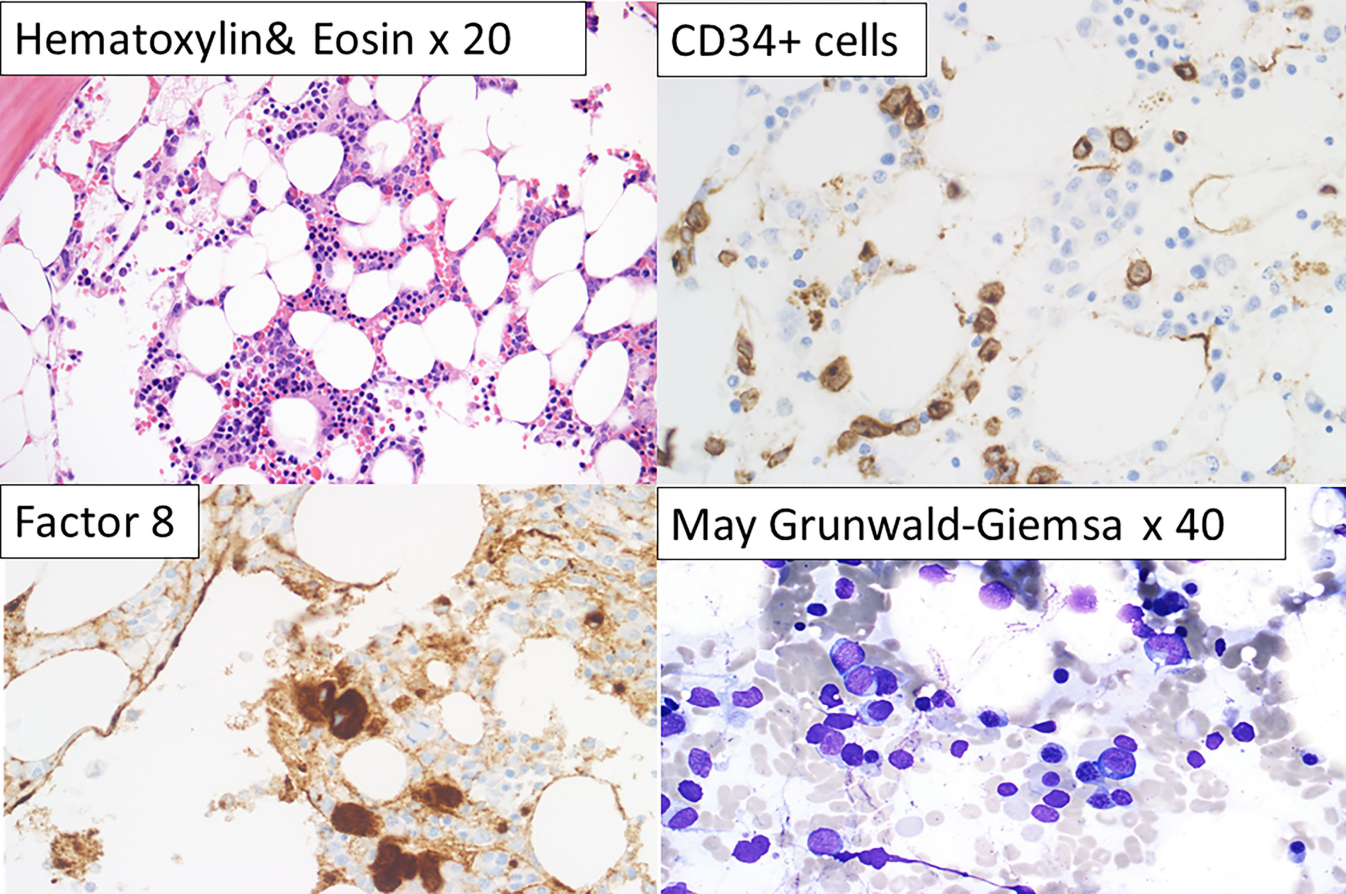
Characteristic bone marrow features of DDX41-m AML. Slightly hypo cellular bone marrow for the patient’s age with relative erythroid hyperplasia, granulocytic and megakaryocytic hypoplasia (Hematoxylin and Eosin, x 20). Small clusters of CD34+ cells (x63). Factor 8 positive normal and small megakaryocytes (x63). Bone marrow particle showing erythroid cells and blasts (May-Grunwald x40).

We reviewed the bone marrow (BM) features of the *DDX41* mutated AML, MDS and MPN patients. Morphologic features seen in the DDX41-s (AMLs and MPNs) were heterogeneous and did not have any distinctive hallmarks differentiating them from non-mutated *DDX41* AMLs or MPNs seen at our institution. Unlike the DDX41-s, the DDX41-m presented with variable degrees of pancytopenia at presentation and no or rare circulating blasts. This is in contrast to DDX41-s AMLs where circulating blasts were seen, and occasionally at high levels. The majority of DDX41-m AMLs were classified as AML NOS and AML MRC, the latter often secondary to MDS (**Table 1**). BM of DDX41-m AMLs were hypo- to normocellular often with relative erythroid hyperplasia and granulocytic and megakaryocytic hypoplasia. Blasts were small, without distinctive morphologic characteristics and at the lower limit for the diagnosis of AML (i.e. in the 20-30% range). The presence of dysplastic changes varied but were frequently minimal, not always meeting the morphologic criteria for MDS (**Figure 4**).

**Figure 5:**
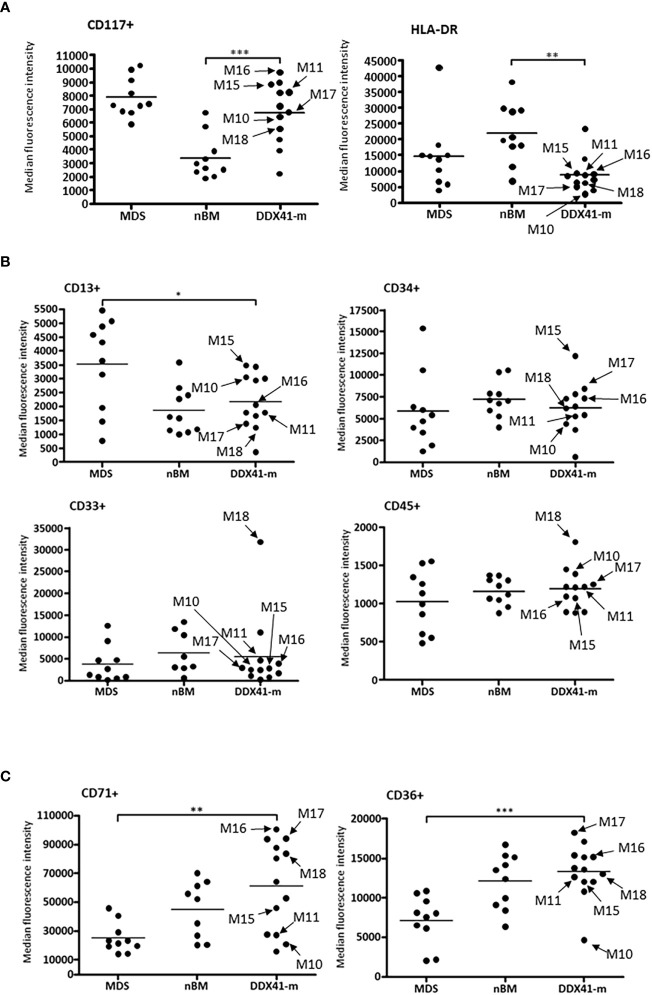
Antigen expression profiles of nucleated red cells and CD34 + cells. Arrows identifying specific DDX41-m discussed in the manuscript. **(A)**. Median expression patterns of CD117 and HLA-DR on CD34 + cells of DDX41-m AML, MDS and nBM. Highest to lowest CD117 expression values for DDX41-m: M16, M9, M6, M15, M8, M11, M20, M17, M10, M18 and M5. Highest to lowest HLA-DR expression values for DDX41-m: M20, M6, M15, M11, M16, M9, M8, M18, M17, M10 and M5. **(B)**. Median expression patterns of CD13, CD33, CD34 and CD45 on CD34 + cells of DDX41-m AML, MDS and nBM. Highest to lowest CD13 expression values for DDX41-m: M15, M19, M10, M20, M6, M16, M8, M11, M17, M18 and M5. Highest to lowest CD33 expression values for DDX41-m: M18, M20, M11, M16, M15, M17, M6, M8, M10, M9 and M5. Highest to lowest CD34 expression values for DDX41-m: M15, M17, M8, M16, M20, M6, M18, M11, M10, M9 and M5. Highest to lowest CD45 expression values for DDX41-m: M18, M6, M10, M17, M8, M11, M20, M16, M15, M5 and M9. **(C)**. Median expression patterns of CD36 and CD71 on nucleated red cells of DDX41-m AML, Myelodysplastic syndrome (MDS) and normal BM (nBM). Highest to lowest CD71 expression values for DDX41-m: M16, M6, M17, M20, M18, M8, M5, M15, M11, M10, M9 and M19. Highest to lowest CD36 expression values for DDX41-m: M17, M20, M16, M19, M8, M5, M18, M11, M15, M9, M6 and M10. *p-value ≤0.05; **p-value ≤0.01; ***p-value ≤0.001.

**Figure 6:**
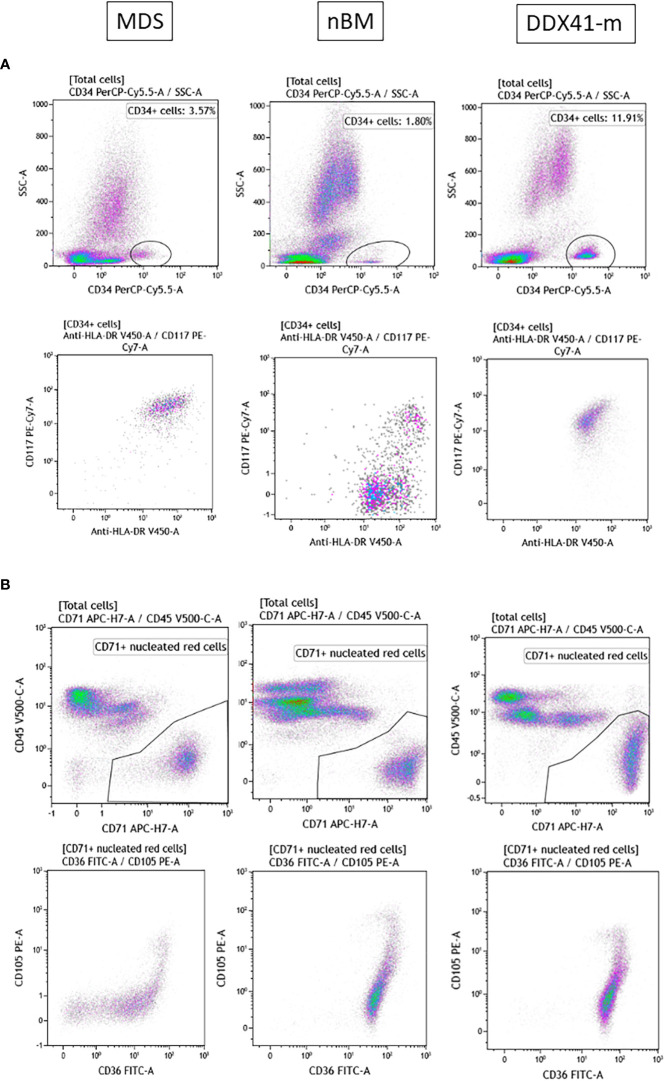
Representative FACS plot of the antigen expression profiles of nucleated red cells and CD34 + cells. MDS. Myelodysplastic syndrome. nBM. Normal BM. DDX41-m AML/MDS. MDS/AML with multiple DDX41 mutations. **(A)**.CD34+ cells are gated on a CD34+ and SSC dot-plot. Expression of CD117 and HLA-DR on CD34+ cells. **(B)**. CD71+ Nucleated red cells are gated on a CD45 and CD71 dot-plot. Expression of CD36, CD71 and CD105.

Given that DDX41-m AML present with clinicopathologic characteristics reminiscent of MDS/AML, we sought to explore whether the immunophenotype of the DDX41-m AML has common features with MDS with excess blasts. We performed an immunophneotpic analysis to look for changes associated with MDS and compared the antigen expression profiles with those of patients with MDS with excess blasts. BM samples of patients with non-hematologic malignancies served as normal controls. We analyzed 12 DDX41-m (cases M5-M6, M8-M11, M15-M20) on which suitable BM sample could be obtained our investigations, 10 normal controls and 10 MDS samples and predominantly focused our analysis on CD34+ progenitor cells and nucleated red blood cells often showing aberrant immunophenotypes in MDS (**Figures 5**, **6**). CD34+ cells were characterized by decreased expression of CD117 and HLA-DR compared to the expression on normal CD34+ cells but similar to that of CD34+ cells of MDS (**Figure 5A**). We did not find consistent changes in the expression of the myeloid markers CD13, CD33 and CD45 (**Figure 5B**). Of interest, the CD34+ cells did not express mature myeloid markers including CD11b CD36 and CD64 (not shown). CD34+ B cells precursors were often <5% of total CD34+ cells. Next, we examined the expression of CD36 and CD71 on nucleated red cells because of their frequent aberrant expression in MDS. Unlike nucleated red cells of MDS, the expression of CD36 and CD71 was normal with similar expression patterns as observed in normal BM samples (**Figure 5C**). Maturing granulocytes and monocytes showed normal maturation patterns.

## Discussion

We applied a targeted myeloid NGS panel to 1930 patients with hematologic malignancies seen at our institution and identified 51 cases (13 MPNs and 38 MDS/AMLs) carrying at least one mutation in *DDX41*. The 2.6% incidence for *DDX41* AML/MDS and MPN patients identified in this study is comparable to the pooled incidence of 3.3% reported in a systematic review of 20 studies on *DDX41* mutated myeloid neoplasms (7). As shown by others (1–9), the majority of DDX41-MDS/AMLs (22 out of 38) harbored multiple (somatic combined to germline) *DDX41* variants (DDX41-m, cases M1-M22); but of note, we also identified 16 MDS/AMLs (cases S14-S29) with a single *DDX41* variant (DDX41-s). In contrast to the MDS/AMLs, all the MPNs (S1-S13) in our study carried a single *DDX41* variant, consistent with previous reports of solitary *DDX41* mutation in DDX41-MPNs (3, 20). Of interest, most of the DDX41-MPNs (10 out 13), also harbor co-occurring MPN disease-causing mutations (*JAK2* = 6, *CALR* = 3, *MPL* =1), which correlates with findings of *JAK2*-mutated MPNs in other DDX41-MPNs previously reported (3). Furthermore, the morphologic features seen in these DDX41-MPNs were in line with those typically encountered in corresponding non *DDX41* mutated (eg. *JAK2*, C*ALR* and *MPL*) MPNs. Similar to the DDX41-s MPNs, the origin of AML could be attributed to other myeloid defining alterations than *DDX41* mutations in the majority (9 out of 16) of the DDX41-s AMLs (**Table 1**, **Figure 2**). In keeping with a different AML etiology (eg. mutations in *CEBPA*, *NPM1*), the clinicopathologic features of the DDX41-s AMLs were heterogeneous and differed from what was seen in DDX41-m AMLs.

In our case series, the underlying cause of AML and MPN could be attributed to well-characterized oncogenic drivers other than a single *DDX41* variant, suggesting that monoallelic *DDX41* variants do not drive the pathophysiology of these myeloid malignancies. For instance, we have identified several DDX41-s that shared the same single *DDX41* variant (eg. p.V303M in S1 and S10, p.M155I in S2 and S19, p.R164W in S20 and S21, p.R159* in S24 and S25) but yet had diverse AML or MPN presentations implying a limited contribution of such solitary *DDX41* variants to the biology of these myeloid malignancies. In keeping with a lesser deleterious potential of monoallelic *DDX41* mutations, the majority of variants identified in the DDX41-s were missense variants located outside of the DEAD box and helicase domain of DDX41 (**Figure 1**, Bottom). This is in contrast to the DDX41-m that have a predominance of loss-of-function variants clustering within the catalytic regions of the encoded protein (**Figure 1**, Top). Of note, even the presence of a single truncating *DDX41* variant, was not sufficient to reproduce hallmarks of MDS/AML seen in the DDX41-m. For example, cases S28 and M3 shared the same truncating p.K381* *DDX41* variant, but have distinct AML presentations at diagnosis (**Table 1**). Similar observations were made in cases S17 versus M2 (same p.I142Sfs*12 variant) or cases S23 versus M15 (same p.G218D variant). In all three scenarios, the difference of AML biology in the DDX41-s versus DDX41-m was attributed to the co-occurrence of a secondary disruptive *DDX41* variant (i.e p.R525H). These findings are in keeping with the preserved normal hematopoiesis demonstrated in mice with monoallelic *DDX41* mutations. In fact, functional analyses showed that hematopoietic defects in these mice were only elicited in the context of biallelic *DDX41* dysfunction (23).

As described by others, the DDX41-m MDS/AMLs from this study were predominantly males of older age (1–9). Contrasting with the DDX41-s (MPNs and AMLs), the origin of AML in the DDX41-AMLs could not be associated to mutations in other myeloid defining biomarkers. Instead, we have encountered several DDX41-m AMLs (eg. M8, M11, M15 and M21) with a normal karyotype and no other myeloid genes variants than the two *DDX41* variants identified, suggesting that biallelic *DDX41* disruption is implicated in the pathophysiology of AML in these individuals. In keeping with a *DDX41* driven AML biology, the DDX41-m AMLs shared common features that are not observed in the DDX41-s AMLs or other non DDX41 mutated AMLs (**Figure 5**). As reported by others, DDX41-AMLs have variable degrees of pancytopenia at presentation and no or rare circulating blasts and variable degrees of uni- and multi-lineage dysplasia, often not meeting the morphologic criteria of MDS (1–9) (**Figure 4**). In keeping with the morphologic findings, the major myeloid lineages including nucleated red cells, maturing granulocytes and monocytes do not show unequivocal changes in their antigen expression (**Figures 5**, **6**). In contrast, the CD34+ myeloid cells have immunophenotypic characteristics akin to MDS CD34+ myeloid cells. The lack of CD36 and CD64 expression and decreased expression of CD117 and HLA-DR suggest a maturation/differentiation block in DDX41-m AML similar to high-grade MDS.

Having found that DDX41-m shared distinct BM immunophenotypic hallmarks that are not observed in DDX41-s, we sought to explore possible correlations with biallelic *DDX41* dysfunction. We thus studied the impact of ten previously uncharacterized missense and in-frame VUSs on *DDX41* function. Previously, we demonstrated that the somatic p.R525H hotspot variant is unable to rescue cellular growth and normal cell cycle in *DDX41* deficient hematopoietic cells (15). Here using a similar experimental approach, we showed that p.R525H also fail to restore growth and cell cycle in HEK293T cells, within which the endogenous *DDX41* expression was repressed using a 3’UTR shRNA (**Figure S1**). Similar to p.R252H, *DDX41* impairment was also seen for another common loss-of-function *DDX41* variant (p.Met1)? (2), further reinforcing the validity of the HEK293T cellular paradigm for our functional analyses.

Using the HEK293T model, we have identified four variants (p.G218D, p.T320I, p.S363del and p.R479W) that recovered between 56% and 66% of HEK293T cells, compared to HEK293T cells transfected with wild-type *DDX41* (blue bars, **Figure 3A**). For reference, similar experiments using the common disruptive *DDX41* p.R525H allele in hematopoietic cells (15, 22) yielded 61% of HEK293T cells. We have also encountered six additional variants (p.A11T, p.M155I, p.P213R, p.E345K, p.A346P and p.D570G) that recouped between 78% and 93% of HEK293T cells compared to HEK293T cells transfected with wild-type *DDX41* (orange bars, **Figure 3A**). Overall, the same *DDX41* mutants also impaired normal cell cycle with a trend towards an accumulation of cells in the G2/M phase (**Figures 3B, C**). We have previously demonstrated cell cycle arrest in G2/M to be a hallmark of *DDX41* dysfunction in hematopoietic cells and to correlate with reduced cellular proliferation. In keeping with these observations, our results suggest that defective cell cycle and ineffective cellular proliferation in HEK293T cells are at least partly due to loss of *DDX41* activity caused by the mutant alleles investigated herein (22). Features of DDX41-m persisted, even in patients showing lesser deficits on HEK293T proliferation. For instance, in case M17 harboring the p.E345K and p.A346P variants (HEK2963T cells count: p.E345K = 88% and p.A346P = 78%, respectively), the expression profile of early myeloid progenitors and/or markers of dyserythropoiesis (**Figure 5**) were comparable to that seen in patient M15 with the p.G218D and p.R525H alleles (HEK2963T cells count: p.G218D = 63% and p.R525H = 61%, respectively).

In this study, we have unraveled the functional role of ten previously uncharacterized *DDX41* variants. Together, evidence gathered on these variants are in favor of a deleterious impact on DDX41 activity. Notable examples include p.G218D, p.T320I, p.E345K, p.A346D, p.S363del and p.R479W.

The p.G218D variant was detected in two DDX41-AMLs (one DDX41-s and one DDX41-m) in our study cohort (**Table 1**, **Figure 2**). This variant was also previously identified in another DDX41-s AML (5) and two additional patients in the context of familial predisposition to hematologic malignancies (Clinvar). The p.G218D variant is very rarely seen in control individuals from the genome aggregation database (gnomAD) as shown by the minor allele frequency (MAF) of 0.0017% (5/282742 alleles). This variant is located within the catalytic DEAD box of DDX41 and is predicted to affect protein function by *in sillico* prediction algorithms (MutationTaster, LRT, Polyphen-2, PROVEAN, SIFT). The p.G218D variant was previously reported as VUS (one submitter) and likely pathogenic (one submitter), in the context of familial predisposition to hematologic malignancies (Clinvar). Functional analyses conducted herein show that this variant impair cellular proliferation and cell cycle in HEK293T cells (**Figure 3**). Furthermore, BM evaluation of patient M15 harboring p.G218D combined to p.R525H showed that this patient shared similar hallmarks to other DDX41-m and suggests biallelic *DDX41* dysfunction (**Figure 5**).

The p.T320I variant was detected in one DDX41-m AML in our study cohort (**Table 1**, **Figure 1**). This variant was also previously identified in another patient in the context of familial predisposition to hematologic malignancies (Clinvar), where it has been classified as a VUS (Clinvar). The p.T320I variant was not previously seen in control individuals from gnomAD. This variant is located within the catalytic DEAD box of DDX41 and is predicted to affect protein function by *in sillico* prediction algorithms (MutationTaster, LRT, Polyphen-2, PROVEAN, SIFT). Functional analyses conducted herein show that this variant impair cellular proliferation and cell cycle in HEK293T cells (**Figure 3**). BM features of patient M18 harboring p.T320I combined to p.G530D showed similar hallmarks to patient M15 and other DDX41-m, in keeping with biallelic *DDX41* dysfunction (**Figure 5**).

The p.E345K variant was detected in one DDX41-m AML in our study cohort (**Table 1**, **Figure 1**). This variant was also identified in another patient in the context of familial predisposition to hematologic malignancies (Clinvar), where it has been classified as a VUS (Clinvar). Furthermore, one DDX41-m AML from this study (**Figure 1**) as well as and two other patients (one AML and one MDS with excess blast) harboring a different missense variant (p.E345D) affecting the same E345 residue have been identified. In both of these external cases, E345D was detected in combination to a germline frameshift *DDX41* variant (3, 4). The p.E345K was not previously seen in control individuals from gnomAD. This variant is located within the catalytic DEAD box of DDX41 and is predicted to affect protein function by *in sillico* prediction algorithms (MutationTaster, LRT, Polyphen-2, PROVEAN, SIFT). Functional analyses conducted herein show that this variant impair cellular proliferation and cell cycle in HEK293T cells (**Figure 3**).

The p.A346P variant was detected in one DDX41-m AML in our study cohort (**Table 1**, **Figure 1**). Two other patients (one AML and one MDS/AML) harboring a different missense variant (p.A346T) affecting the same A346 residue have been identified. In both cases, A346T was detected in combination to another deleterious DDX41 alleles (germline p.Met1? variant and p.R525H) (3, 5). The p.A346P variant was not previously seen in control individuals from gnomAD. This variant is located within the catalytic DEAD box of DDX41 and is predicted to affect protein function by *in sillico* prediction algorithms (MutationTaster, LRT, Polyphen-2, PROVEAN, SIFT). Functional analyses conducted herein show that this variant results in impairment of *DDX41* activity on cellular proliferation and cell cycle in HEK293T cells (**Figure 3**). Of note, the BM features of patient M17 harboring p.A346P combined to p.E345K (see above) were suggestive of biallelic DDX41 dysfunction as seen with other DDX41-m investigated herein (**Figure 5**).

The p.S363del variant was detected in one DDX41-m AML in our study cohort (**Table 1**, **Figure 2**) as well as another DDX41-m AML (4). The p.S363del variant is very rarely seen in control individuals from gnomAD as shown by with a minor allele frequency (MAF) of 0.00039% (1/251030 alleles). This variant affects the catalytic DEAD box of DDX41. Functional analyses conducted herein show that this variant impair cellular proliferation and cell cycle in HEK293T cells (**Figure 3**).

The p.R479W variant was detected in one DDX41-s MPN in our study cohort (**Table 1**, **Figure 2**). This variant was also previously identified in another DDX41-s that presented with MDS (4). Three other patients (one AML, one MDS and one MDS/MPN) harboring a different missense variant (p.R479Q) affecting the same R479 residue have been reported (4). The p.R479W is very rarely seen in control individuals from the genome aggregation database (gnomAD) as shown by with a minor allele frequency (MAF) of 0.0016% (4/251358 alleles). This variant is located within the catalytic helicase domain of DDX41 and is predicted to affect protein function by *in sillico* prediction algorithms (MutationTaster, LRT, Polyphen-2, PROVEAN, SIFT). Functional analyses conducted herein show that this variant impair cellular proliferation and cell cycle in HEK293T cells (**Figure 3**).

In this study, we have identified loss-of-function (eg. nonsense, frameshift, splicing) *DDX41* variants as well as disruptive *DDX41* alleles from the functional analyses conducted. The nature of individual variants (eg. frameshift versus missense) did not seem to determine patient outcome. Rather, the combination of two disruptive *DDX41* variants was associated with similar clinical pathologic findings and an overall favorable AML outcome compared to other AML entities. Our study showed evidence of biallelic *DDX41* disruption in 17 out of the 22 DDX41-m (cases M1-M17) as highlighted by the combination of at least two deleterious variants (**Figure 2**). These include cases M1-M5 (carrying two loss-of-function *DDX41* variants), cases M6-M13 (carrying a loss-of-function variant combined to a disruptive *DDX41* allele in HEK293T cells) and cases M14 and M17 (carrying two disruptive *DDX41* alleles in HEK293T cells). Of note, from short reads NGS data, we were able to confirm that the deleterious *DDX41* variants in cases M5 (p.R525H and p.T529Rfs*12), M10 (p.R525H and p.F535Nfs*6) and M17 (p.E345K and p.A346T) were in *trans* configuration, further corroborating biallelic *DDX41* disruption. Furthermore, BM morphology as well as immunophenotype of the DDX41-m investigated herein were similar and distinct from pathophysiologic findings in DDX41-s, MDS or normal bone marrows (**Figures 5**, **6**). Unique hallmarks seen in these DDX41-m and not the DDX41-s are in keeping with findings that defective hematopoiesis only occur in the context of biallelic *DDX41*dysfunction (23).

As shown by others, DDX41-m typically involve a germline and a somatic *DDX41* variant. However, in this study, we have also identified two individuals harboring two somatic deleterious *DDX41* alleles (eg. M10: p.R525H at 15.9% and p.F535Nfs*6 at 12.4%; M20: p.R252H at 7% and p.G587C at 5.9%) indicating that biallelic DDX41 disruption can also occur in the context of two somatic variants. Although rare (4 out of 277), biallelic somatic variants (based on VAF< 10%) were previously reported in individuals having MDS with excess blasts (6). Implications of such biallelic somatic variants are multifold. First, testing methodologies should be adapted to improve the detection of the somatic *DDX41* variants that are commonly encountered at low levels (eg. in this study average VAF: 9.5%, minimum VAF: 3.2%). Second, cases with two somatic *DDX41* variants share similar clinicopathologic findings to other DDX41-m AMLs with a germline and a somatic *DDX41* allele and as such should be managed accordingly. Third, unlike other DDX41-m AMLs, germline testing is not warranted in the context of two somatic *DDX41* alleles are detected.

In summary, we have studied the genomic and BM features of 51 patients with *DDX41* mutations. Of these, only individuals with two DDX41 variants shared similar clinicopathologic findings as described by others (1–9). Here, we also observed that unlike individuals with monoallelic *DDX41* alterations, patients harboring two DDX41 alleles present with common BM morphology and immunophenotypic hallmarks. As highlighted by others, DDX41-m AMLs have a more favorable outcome than seen in other AML entities; thus, accurate identification and interpretation of DDX41-m variants, particularly germline mutations, are of crucial importance to clinical management of affected individuals and genetic counselling of at risk family members. In this study, we have determined the deleterious potential of ten previously uncharacterized *DDX41* variants. Here, we have corroborated genomic and clinicopathologic findings on *DDX41* mutated MDS/AML (1–9). Our work contributes to expanding knowledge on these DDX41 related malignancies with a particular emphasis on biallelic *DDX41* disruption and deleterious *DDX41* alleles unraveled herein. Furthermore, pathologic hallmarks of DDX41-m evaluated in this study can serve as a basis for molecular testing in patients with suspicion of biallelic *DDX41* dysfunction. More extensive studies including such as single cell RNA sequencing will help shed more light on the biological implication of DDX41 in the pathophysiology of AML at a cellular level.

## Data availability statement

The data presented in the study is deposited in the ClinVar repository, accession number SUB13174259.

## Ethics statement

The studies involving human participants were reviewed and approved by UHN research and ethics board. Written informed consent for participation was not required for this study in accordance with the national legislation and the institutional requirements.

## Author contributions

J-MC-C, AT and HM designed and supervised the experiments conducted in this study. DM, HS, KY, ASc, DK and MM were involved in the clinical management of patients and contributed to samples collection. J-MC-C, MC and AS conducted molecular analyses on the patients. ASm conducted cytogenetic analyses. AT and EK performed tumor assessment on the patients. AT and MM acquired clinicopathological findings on the patients. SS and HM performed functional validation experiments. J-MC-C, MM, AT and HM wrote the manuscript. All authors contributed to the article and approved the submitted version.
