# Supplementary material for: Biallelic disruption of DDX41 activity is associated with distinct genomic and immunophenotypic hallmarks in acute leukemia
Source: Front Oncol. 2023 Jun 26;13:1153082. doi: 10.3389/fonc.2023.1153082 (PMC10331015; doi:10.3389/fonc.2023.1153082)
Supplement: Supplementary file 1 [file Image_1.pdf]

Supplementary Material

Article Title

Anne Tierens [1], Elizabeth Kagotho [2], Satoru Shinriki [3], Andrew Seto [4], Adam C. Smith [1,4], Melanie Care [4], Dawn Maze [5], Hassan Sibai [5], Karen W. Yee [5], Andre C Schuh [5], Dennis Dong Hwan Kim [5], Vikas Gupta [5], Mark D. Minden [5], Hirotaka Matsui [3], José-Mario Capo-Chichi [1,4*]

1. Department of Laboratory Medicine and Pathobiology, University of Toronto, Toronto, Canada
2. Department of Pathology and Laboratory Medicine, Aga Khan University Hospital, Nairobi, Kenya
3. Department of Molecular Laboratory Medicine, Faculty of Life Sciences, Kumamoto University, Japan
4. Division of Clinical Laboratory Genetics, Laboratory Medicine Program, University Health Network, Toronto, Canada
5. Department of Medicine Medical Oncology and Hematology, University of Toronto, Princess Margaret Cancer Centre, Toronto, Canada

*** Correspondence:**
Corresponding Author
jose-mario.capo-chichi@uhn.ca

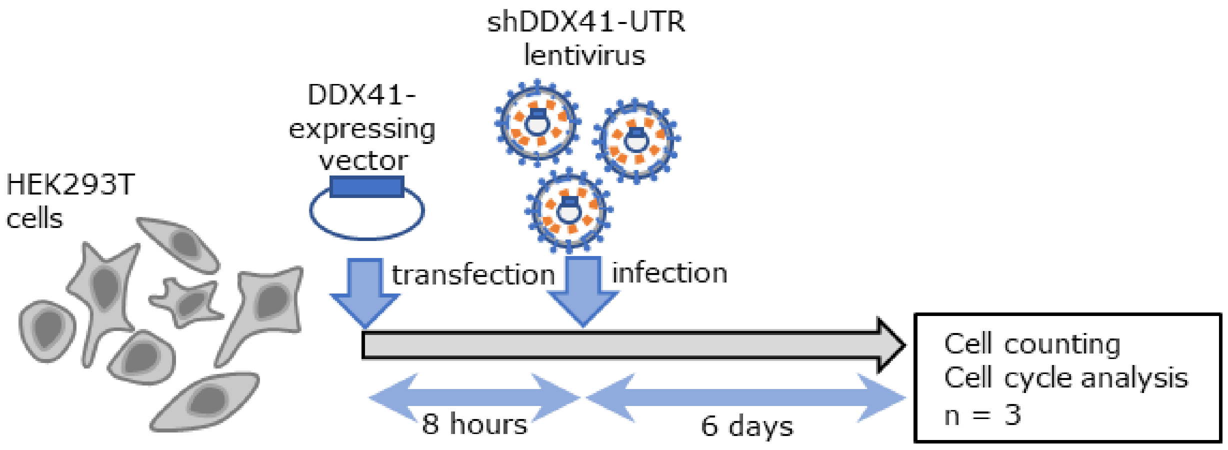

Figure S1. **Schematic representation of DDX41 experiments in HEK293T cells**. HEK293T was transfected with plasmid DNA containing cDNA of a DDX41 variant and then infected with a lentivirus that express shRNA targeting the 3’UTR of DDX41. Number of cells and cell cycle status were analyzed 6 days after the transfection.
