# Supplementary material for: Biallelic disruption of DDX41 activity is associated with distinct genomic and immunophenotypic hallmarks in acute leukemia
Source: Front Oncol. 2023 Jun 26;13:1153082. doi: 10.3389/fonc.2023.1153082 (PMC10331015; doi:10.3389/fonc.2023.1153082)
Supplement: Supplementary file 2 [file Table_1.pdf]

Supplementary Material

Article Title

Anne Tierens [1], Elizabeth Kagotho [2], Satoru Shinriki [3], Andrew Seto [4], Adam C. Smith [1,4], Melanie Care [4], Dawn Maze [5], Hassan Sibai [5], Karen W. Yee [5], Andre C Schuh [5], Dennis Dong Hwan Kim [5], Vikas Gupta [5], Mark D. Minden [5], Hirotaka Matsui [3], José-Mario Capo-Chichi [1,4*]

1. Department of Laboratory Medicine and Pathobiology, University of Toronto, Toronto, Canada
2. Department of Pathology and Laboratory Medicine, Aga Khan University Hospital, Nairobi, Kenya
3. Department of Molecular Laboratory Medicine, Faculty of Life Sciences, Kumamoto University, Japan
4. Division of Clinical Laboratory Genetics, Laboratory Medicine Program, University Health Network, Toronto, Canada
5. Department of Medicine Medical Oncology and Hematology, University of Toronto, Princess Margaret Cancer Centre, Toronto, Canada

*** Correspondence:**
Corresponding Author
jose-mario.capo-chichi@uhn.ca

Table S1. Custom next-generation sequencing somatic myeloid panel.

CCDS. Complete consensus DNA sequence. Gene specific targets were designed using baits from Oxford Gene Technologies (OGT).

Genes	Transcript	Target
ASXL1	NM_015338.5	exon 13
BCOR	NM_001123385.1	full CCDS
BCORL1	NM_021946.4	full CCDS
BRAF	NM_004333.4	exon 15
CALR	NM_004343.3	exon 9
CBL	NM_005188.3	exons 8,9
CEBPA	NM_004364.3	full CCDS
CSF3R	NM_156039.3	exons 14-17
CTNNA1	NM_001903.3	full CCDS
CUX1	NM_001202543.1	full CCDS

**DDX41**	**NM_016222.3**	**full CCDS**
DNMT3A	NM_022552.4	full CCDS
ETNK1	NM_018638.4	full CCDS
ETV6	NM_001987.4	full CCDS
EZH2	NM_004456.4	full CCDS
FBXW7	NM_033632.3	exons 9-11
FLT3	NM_004119.2	exons 14,15,20
GATA2	NM_032638.4	exons 2-6
GNAS	NM_000516.4	exons 8,9
IDH1	NM_005896.3	Exon 4
IDH2	NM_002168.3	Exon 4
IKZF1	NM_006060.5	full CCDS
IRF1	NM_002198.2	full CCDS
JAK1	NM_001321853.1	full CCDS
JAK2	NM_004972.3	exons 12,14
KIT	NM_000222.2	exons 2, 8-11, 13,17
KMT2A	NM_001197104.1	full CCDS
KRAS	NM_033360.3	exons 2,3
MPL	NM_005373.2	exon 10
MYD88	NM_002468.4	exons 3,5
NOTCH1	NM_017617.3	exons 26-28, 34
NPM1	NM_002520.6	exon 12
NRAS	NM_002524.3	exons 2,3
PAX5	NM_016734.2	full CCDS
PHF6	NM_032458.2	full CCDS
PPM1D	NM_003620.3	full CCDS
PTPN11	NM_002834.3	exons 3,13
RAD21	NM_006265.2	full CCDS
RUNX1	NM_001754.4	full CCDS
SETBP1	NM_015559.2	exon 4
SF3B1	NM_012433.2	exons 13-16
SH2B3	NM_005475.2	full CCDS
SRSF2	NM_001195427.1	exon 1
STAG2	NM_001042749.2	full CCDS
TET2	NM_001127208.2	exons 3-11
TP53	NM_000546.5	exons 2-11
U2AF1	NM_001025203.1	Exons 2,6
WT1	NM_024426.3	exons 7,9

ZRSR2	NM_005089.3	full CCDS
