# Supplementary material for: Biallelic disruption of DDX41 activity is associated with distinct genomic and immunophenotypic hallmarks in acute leukemia
Source: Front Oncol. 2023 Jun 26;13:1153082. doi: 10.3389/fonc.2023.1153082 (PMC10331015; doi:10.3389/fonc.2023.1153082)
Supplement: Supplementary file 3 [file Table_2.pdf]

Supplementary Material

Article Title

Anne Tierens [1], Elizabeth Kagotho [2], Satoru Shinriki [3], Andrew Seto [4], Adam C. Smith [1,4], Melanie Care [4], Dawn Maze [5], Hassan Sibai [5], Karen W. Yee [5], Andre C Schuh [5], Dennis Dong Hwan Kim [5], Vikas Gupta [5], Mark D. Minden [5], Hirotaka Matsui [3], José-Mario Capo-Chichi [1,4*]

1. Department of Laboratory Medicine and Pathobiology, University of Toronto, Toronto, Canada
2. Department of Pathology and Laboratory Medicine, Aga Khan University Hospital, Nairobi, Kenya
3. Department of Molecular Laboratory Medicine, Faculty of Life Sciences, Kumamoto University, Japan
4. Division of Clinical Laboratory Genetics, Laboratory Medicine Program, University Health Network, Toronto, Canada
5. Department of Medicine Medical Oncology and Hematology, University of Toronto, Princess Margaret Cancer Centre, Toronto, Canada

*** Correspondence:**
Corresponding Author
jose-mario.capo-chichi@uhn.ca

Table S2. Primer pairs used to construct DDX41 expression vectors.

Lower case bases in forward primers indicate mutations used to construct DDX41 variants.

Variant (cDNA)	Variant (protein)	Forward primer	Reverse primer
c.3G>A	p.? (p.M1I)	aGAGGAGTCGGAACCCGAACGGAAG	ATGGTGGCGAATTCCACCACACTGG
c.31G>A	p.Ala11Thr	aCTCGCACCGACGAGGTGCCTGCCG	CCGCTTCCGTTCGGGTTCCGACTCC
c.465G>A	p.Met155Ile	aTCTGAAGAGCGACATGAGCGCGTG	ATGCTCAGAACATAACGGGGTGGAG
c.638C>G	p.Pro213Arg	gCACCATTCTATCTGGCCGTGACATG	GGATGCCCTGGATCTGAATGGGTG
c.653G>A	p.Gly218Asp	aCCGTGACATGATAGGCATCGCTTTC	CAGATAGAATGGTGGGGATGCCCTG
c.959C>T	p.Thr320Ile	tCCCGGGGCGCCTCATGGATTTGCTG	TGGCCACCATCATGTGTACACCGTG
c.1033G>A	p.Glu345Lys	aAGGCTGACCGCATGATCGACATGG	GTCCAGGGCCAGGTAGCGACAGATG
c.1036G>C	p.Ala346Pro	cCTGACCGCATGATCGACATGGGC	CTCGTCCAGGGCCAGGTAGCGACAG
c.1088_1090delCCT	p.Ser363del	ACTTCAAGGGCCAGCGACAGACCC	AGAAGATGGTACGGATGTCACCCTC
c.1435C>T	p.Arg479Trp	tGGGAGGGCAAGAAGGATGTCCTAG	GAATGCCTCGATGGCCTTAGTCCG

c.1574G>A	p.Arg252His	GATTGGCCGCACCGGGCaCTCGGGAAACAC	GTGTTTCCCGAGtGCCCGGTGCGGCCAATC
c.1709A>G	p.Asp570Gly	gTGAGTCCATGCTGGACATTGGAGG	CCCCGCAATGCAGCACCTGCAGCAC

frontiers
